# Combined Ionizing Radiation Caused Cognition and Non-Cognition Behavior Benefits and Modulated Microglial Activity in Wild-Type and Alzheimer’s-like Transgenic Mice

**DOI:** 10.3390/biology14060682

**Published:** 2025-06-11

**Authors:** Viktor S. Kokhan, Anna I. Levashova, Maxim S. Nesterov, Vladimir A. Pikalov, Maria M. Chicheva

**Affiliations:** 1 V.P. Serbsky National Medical Research Centre for Psychiatry and Narcology, 119034 Moscow, Russia; 2 Scientific Center of Biomedical Technologies of the Federal Medical and Biological Agency of Russia, 143442 Settlement Svetlye Gory, Russia; info@scbmt.ru (A.I.L.);; 3 Institute for High Energy Physics Named by A.A. Logunov of NRC “Kurchatov Institute”, 142281 Protvino, Russia; pikalov@ihep.ru; 4 Institute of Physiologically Active Compounds of the Russian Academy of Sciences, 142432 Chernogolovka, Russia; chicheva.mariya@gmail.com

**Keywords:** ionizing radiation, carbon-12, gamma-rays, neuroinflammation, Alzheimer’s disease, tauopathy, anxiety, cognitive abilities, cytokines

## Abstract

Alzheimer’s disease (AD) is one of the primary causes of disability and dependency among aging populations worldwide. Neuroinflammation may be a potential therapeutic target in AD. Moreover, ionizing radiation may be a tool for modulating neuroinflammation. Here, we used three mouse lines—C57Bl/6 and the transgenic AD models 5xFAD and Tau P301S—to investigate the effects of combined ionizing radiation (γ-rays and carbon-12 nuclei) on emotional state, cognitive abilities, and markers of microglial activation. The obtained data show that combined irradiation results in enhanced exploratory behavior and spatial learning in the C57Bl/6 mice. The same changes, as well as a decrease in anxiety, were found in the Tau P301S mice. Irradiation of the 5xFAD mice resulted in improved welfare and ability to discriminate odors. At the same time, irradiation led to an increase in the level of pro- and anti-inflammatory cytokines in the prefrontal cortex and, to a lesser extent, in the hippocampus of the C57Bl/6 and Tau P301S mice. An increase in macrophage inflammatory protein-1α in the prefrontal cortex and a decrease in interleukin 2β in the hippocampus were found in the 5xFAD mice. Taken together, our data indicate that ionizing radiation exposure is an adequate tool to modulate microglial activity in the brain and may provide cognitive and non-cognitive behavioral benefits in neurodegenerative disease conditions.

## Simple Summary

There is currently no effective treatment for Alzheimer’s disease (AD). Neuroinflammation is considered one of the promising targets for the treatment of AD and other proteinopathies. Moreover, several studies have suggested that ionizing radiation (IR) could be an effective method for targeting neuroinflammation. In this study, we examined the effects of combined IR (gamma rays and high-energy carbon-12 nuclei) on AD-related behavioral symptoms and cytokine content in the prefrontal cortex and hippocampus of 5xFAD and Tau P301S mice lines (transgenic models of AD), as well as naïve C57Bl/6 mice. The results showed that IR exposure resulted in cognitive and non-cognitive behavioral benefits in all mouse lines used. Alongside this, the C57Bl/6 and Tau P301S irradiated mice showed an increase in cytokine content predominantly in the prefrontal cortex. In contrast, the 5xFAD mice showed a limited “re-balancing” effect of IR exposure on cytokine content. Thus, the results indicate the potential use of combined radiotherapy in the treatment of AD.

## 1. Introduction

Neurodegenerative diseases are a group of heterogeneous disorders that challenge healthcare systems, particularly in countries with aging populations. Alzheimer’s disease (AD) is the most common neurodegenerative disease and is often associated with memory deficits and cognitive decline. An estimated 6.2 million Americans aged 65 and above are living with AD today [1]. In Russia, there are no official statistics on AD, but according to various estimates, ~1.8 million people may be affected. There is no cure for Alzheimer’s disease, and existing treatments can only slow the progression of the disease and alleviate symptoms [2,3].

*Inter alia*, AD is associated with dysregulation in the innate immune system and uncontrolled inflammatory processes. However, the exact mechanisms by which innate immunity influences AD remain elusive [4,5]. The innate immune system, consisting of pattern-recognition receptors, macrophages (microglia cells) and the complement system, is known to play a key role in CNS homeostasis and neuroinflammation. Notably, neuroinflammation appears at an early stage of AD—before the appearance of neurofibrillary pathology and memory decline [6,7]. A number of studies have shown that neuroinflammation can be considered a double-edged sword: on the one hand, inflammation is an integral part of the pathological process in neurodegenerative disease conditions [8,9]; on the other hand, it participates in neuroregeneration, particularly by stimulating the clearance of debris by microglia [10]. Moreover, microglia have been implicated in the immunologic regulation of synaptic plasticity, partly through the production of neurotrophic factors [11] and synaptic pruning [12]. Recent studies show that neuroinflammation may be a potential therapeutic target in AD [3,13]. Moreover, ionizing radiation (IR) may be an effective tool for modulating neuroinflammation via its influence on the innate immune system [14].

A number of studies have revealed anti-inflammatory effects of γ-rays in the hippocampus (HPC) of AD mouse models under different irradiation scenarios: chronically (1 mGy/day, 300 days; apolipoprotein E knockout mice) or fractionated (5 × 0.6 Gy or 5 × 2 Gy; 5xFAD mice) [15,16]. In contrast, in another study, fractionated γ-rays (5 × 2 Gy) induced microglia increase at 4 weeks post-irradiation, reduction of amyloid beta peptide (Aβ) plaques in the HPC, and, importantly, improved spatial learning in the B6.Cg-Tg AD mouse model [17]. These findings were confirmed in an independent study [18]. Irradiation with γ-rays (1.79 Gy) also causes a decrease in another protein involved in AD pathogenesis—microtubule-associated protein tau—in the healthy brain of swine [19]. In addition, several clinical case reports have shown symptom relief in patients with both AD and Parkinson’s disease after exposure to low doses of IR [20].

Much less is known about the effect of heavy ion irradiation on neuroinflammation and the course of neurodegeneration. Irradiation of the healthy brain with ^56^Fe (1–4 Gy, ~175 keV/µm) leads to a dose-dependent pro-inflammatory effect [21]. Notably, brain irradiation of mice with ^56^Fe (2 Gy, ~180 keV/µm) attenuated the lipopolysaccharide-induced inhibition of long-term potentiation at 1 and 3 months, but not at 6 and 12 months post-irradiation [22]. Irradiation with ^12^C (15 keV/µm) at doses of 0.05 or 0.1 Gy did not result in significant accumulation of amyloid-beta precursor, Aβ, tau, or phospho-tau proteins in the hippocampal CA1 region [23]. In contrast, irradiation with 0.1 or 0.5 Gy ^56^Fe (147 keV/µm) led to a decrease in both cerebral Aβ level and microglial activation, but only in female APPswe/PS1dE9 mice, without affecting wild-type animals [24]. In our previous study utilizing a combined irradiation model (0.24 Gy γ-rays and 0.18 Gy ^12^C, 10.3 keV/μm), we found that IR alleviated behavioral symptoms in Tau P301S and 5xFAD mice [25].

IR can also have a neuroprotective/neuroregenerative effect. It has been shown that a local irradiation (10 Gy, X-rays) of the right subventricular zone enhances the proliferation rate and neuroreparation in response to chemically induced demyelination in the striatum of mice [26]. Moreover, IR blocks pro-apoptotic effects and reverses pathological locomotor and anxiety behavior, which is caused by long-term antiorthostatic suspension (ground-based model of microgravity) in various scenarios: 40 mGy γ-rays (chronically for 21 days) or combined γ-rays (0.5 Gy × 6, fractionated) and H^+^ (1.5 Gy, 0.4 keV/μm, acute) [27,28]. In another study, combined IR (0.4 Gy γ-rays and 0.14 Gy ^12^C, 10.3 keV/μm) had a pro-cognitive effect in rats that persisted up to 7 months post-irradiation [29].

Based on these data, we have previously hypothesized that IR exposure at certain doses and compositions may be an effective tool in the treatment of neurodegenerative conditions, including AD [30].

It is well known that the two most widely accepted pathogenic mechanisms of AD include the amyloid and tau hypotheses. The early stages of tau pathology are associated with the reduced functional connectivity in some neocortex regions (including the prefrontal cortex, PFC) and the HPC, and this reduction is associated with memory decline [31]. Relying on the above, we are examining the hypothesis that combined IR exposure (γ-rays and ^12^C) can reverse AD-like behavioral pathologies, and that this effect may be associated with changes in microglial activation in key brain regions for learning and memory—the PFC and HPC. We are using two transgenic mouse lines—5xFAD and Tau P301S, which are modeling Aβ- and tau-pathies, respectively—and naïve mice (C57Bl/6) to evaluate the effects of combined IR on the healthy brain.

## 2. Materials and Methods

### 2.1. Animals

Male mice aged 4 months from the following lines were used in this study: C57Bl/6 (30–33 g weight), hemizygous Tau P301S (22–24 g weight), and hemizygous 5xFAD (23–24 g weight). The 5xFAD [32] and Tau P301S [33] lines were originally obtained from Prof. V. Buchman (Cardiff University, Cardiff, UK). All of the mouse lines used in this study were obtained from the Center for Collective Use of the Institute of Physiologically Active Compounds RAS (Chernogolovka, Russia). Transgenic lines were maintained on a C57BL/6J strain background. Animals were housed in groups of two or three per cage in a standard environment (12 h light/dark cycle, 19–22 °C, and 50–70% relative humidity) with ad libitum food and water.

### 2.2. Experiment Timeline

**Figure 1:**
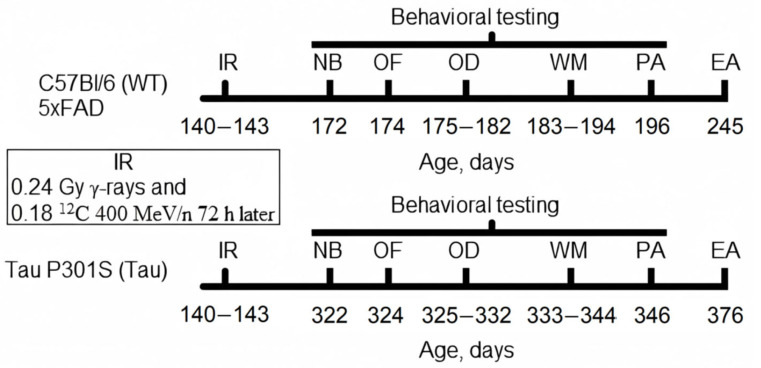
Study timeline. IR—exposure to combined ionizing radiation, NB—nest building test, OF—open field test, OD—odor discrimination test, WM—water maze, PA—passive avoidance test, EA—euthanasia. ^12^C—carbon-12 nuclei, 400 MeV/*n*, linear energy transfer 11 keV/µm. The ages of the mice are shown in days.

Five days before the experiment, all mice were weighed one at a time and distributed into groups according to the minimization approach in randomization, so that animals with the same weight were placed in different groups [34]. The following groups of animals were formed for the experiment: control (WT, *n* = 7) and irradiated (WT + R, *n* = 6) C57Bl/6 mice, control (5xFAD, *n* = 9) and irradiated (5xFAD + R, *n* = 9) 5xFAD mice, and control (Tau, *n* = 11) and irradiated (Tau + R, *n* = 8) Tau P301S mice. The timeline of this study is shown in Figure 1.

### 2.3. Combined Irradiation

Irradiation with γ-quanta (~662 keV) was performed at the GOBO-60 facility equipped with a ^137^Cs source (certified activity of 72 g-eq ^226^Ra). Mice were whole-body irradiated daily (10 mGy/h) at a total absorbed dose of 0.24 ± 0.03 Gy. The absorbed dose was measured with thermoluminescent monocrystalline DTG-4 (LiF-Mg × Ti) detectors (A.P. Vinogradov Institute of Geochemistry SB RAS, Irkutsk, Russia). A Reader Harshaw TLD model 3500 (Thermo Fisher Scientific, Waltham, MA, USA) was used to anneal the detectors and for dose calculation.

Seventy-two hours after γ-ray irradiation, the heads of the mice were irradiated with ^12^C nuclei (400 MeV/*n*, 0.18 ± 0.013 Gy, linear energy transfer 11 keV/μm) in a U-70 charged-particle accelerator (NRC “Kurchatov Institute”—IHEP, Protvino, Russia). Dosimetric monitoring of irradiation was performed using a DKS-AT5350/1 dosimeter (Atomtex, Minsk, Belarus) with a TM30010-1 ionization chamber (PTW-Freiburg, Freiburg, Germany).

### 2.4. Nest Building

The nest building test was performed using an established protocol [35]. Mice were housed individually in clean cages and provided with two pressed cotton squares measuring 5 × 5 cm (Newfarm LLC, Ovcharnoe, Russia). After 24 h, the dimensions of the nest were measured and scored according to the following criteria: 0—materials untouched; 1—torn material; 2—material made flat against the cage bedding; 3—wall height less than 3 cm on average in all four quadrants; 4—wall height 3–5 cm on average in all four quadrants; 5—wall height > 5 cm.

### 2.5. Open Field

A Tru Scan Photo Beam Tracking System (Coulbourn Instruments, Cambridge, MD, USA) with a square box size of 30 × 30 cm was used. The unit was equipped with a floor with holes. Data acquisition and analysis were controlled by Tru Scan v.2.02 software (Coulbourn Instruments, USA). The field was uniformly illuminated at 60 lx. Alternating between groups, a mouse was placed in a corner of the field, and the horizontal and vertical activity scores were recorded for 3 min. After each animal, the floor was wiped with 70% ethanol and dried.

### 2.6. Odor Discrimination

The test was performed according to the methodology published earlier [36], with some differences. In the test, a 60 × 40 × 40 cm box was used. The box was equipped with a rising partition made of opaque plastic (polymethylmethacrylate) positioned in the middle of the long side. On one side of the partition was a starter compartment in which the mouse was placed, and on the other side were two feeders—plastic containers 4 cm in diameter and 1.2 cm high. Birch sawdust was used as a filler, and sweet multigrain cereal (Syrial Partners Rus, Russia) was used as a positive reinforcement (reward). Light mineral oil (320 g/mol, Cat. No. 1443952, USP, North Bethesda, MY, USA) was used as a solvent for odorants. During the preparatory stage, 5% (*v*/*v*) clove and laurel essential oils were used. At the testing stage, S(+) and R(-) enantiomers of carvone (Acros Organics, Belgium) were used as a working pair of odorants, diluted in mineral oil at a ratio of 10:11 (*v*/*v*). The feeder marked with S(+)-carvone did not contain a reward, whereas the one with R(-)-carvone did. The mouse was placed in the starting compartment, and after 15 s, the partition was lifted, determining which feeder’s filler the mouse would start digging in. In the case that the feeder containing the reward was reached first, this trial was considered correct. To explore the learning dynamics, we calculated the learning coefficient (the ratio of correct feeder choices to the total number of trials) over every 5 trials. A total of 20 trials were conducted.

### 2.7. Water Maze

An 80 cm diameter black pool was used. A 6 cm diameter black plastic platform (invisible to the animal) was immersed 1 cm below the water surface, always in the same place. The mouse was released into the water at the edge of the pool at a chosen point and allowed to swim freely for 60 s. The same starting point was used for all mice within one trial. There were two trials per day, with the starting point changing for each trial. Spatial learning continued for 5 days. The probe test was performed after the final learning on day 5: the platform was removed from the pool, the mouse was released into the water at the edge, and the time spent in the quadrant where the platform had been located was recorded for 60 s. One week later, long-term spatial memory was tested using the same scheme as in the learning stage (2 trials).

### 2.8. Step-Down-Type Passive Avoidance

The chamber used in the test (25 × 25 × 45 cm; width × length × height) was equipped with an electrified grid floor. In the center of the grid floor, there was a plastic platform (6 × 6 × 1.2 cm). During the acquisition stage, mice were placed on the platform and covered with a glass (5.5 cm diameter). After 30 s, the glass was removed, and the time it took the mouse to step with all four paws on the electrified grid floor was recorded. Subsequently, a mild electric foot shock (0.3 mA constant current, 100 Hz, meander) was applied for 3 s, and the test was completed. The recall stage was carried out after 24 h, where the time it took for the mouse to step down from the platform with all four paws was recorded, and no electric shock was used. The maximum allowable time for stepping down was 120 s.

### 2.9. Euthanasia and Tissue Preparation

Mice were euthanized by cervical dislocation. Saline perfusion through the carotid artery was performed before brain dissection. The prefrontal cortex (PFC) and hippocampus (HPC) were dissected on a thermoelectric cooling table (+2 °C) and stored in liquid nitrogen.

### 2.10. Cytokines Multiplex Analysis

The multiplex assay was performed using a Bio-Plex Pro Mouse Cytokine 23-Plex kit (Bio-Rad, Hercules, CA, USA). Bead preparation, handling, and plate processing were conducted according to the manufacturer’s protocol. The plates were washed using a Bio-Plex Pro Wash Station (Bio-Rad, Hercules, CA, USA) and read using a Bio-Plex MAGPIX Multiplex Reader (Bio-Rad, Hercules, CA, USA). The concentration of cytokines in the tested samples was automatically determined using standard calibration dilutions and Bio-Plex Manager software v.6.1 for equipment management and initial data processing, followed by Bio-Plex Data Pro software v.1.2 for final data processing (both Bio-Rad, USA). The target protein content was normalized to the total protein content of the sample.

Data on the contents of the following analytes were obtained: interferon-γ (IFNγ), interleukin (IL)-1α, IL-1β, IL-2, IL-3, IL-6, IL-9, IL-10, IL-12(p40), IL-17A, keratinocyte-derived chemokine (KC, CXCL1), macrophage inflammatory protein-1α (MIP-1α) and 1β (MIP-1β), C-C motif ligand 5 (CCL5), C-C motif ligand 11 (CCL11), and tumor necrosis factor-α (TNF-α). The content of IL-4, IL-5, IL-12 (p70), IL-13, G-CSF (granulocyte colony-stimulating factor), GM-CSF (colony-stimulating factor 2), and MCP-1 (monocyte chemoattractant protein 1) was below the detectable level.

### 2.11. Data Analysis

Standard data processing was performed with Statistica v.12 software (StatSoft Inc., Tulsa, OH, USA) and Python v.3.11 with NumPy v.2.2.4 (open-source library). The Shapiro–Wilk test was used to assess the normality of data distribution. Levene’s test was used to verify homoscedasticity between the compared samples. If the level of statistical significance (p) exceeded 0.05 in these tests for the analyzed data, the results were presented as mean ± standard deviation (SD), and parametric analysis methods were used. Conversely, if the significance level was below 0.05, the data were presented as median ± interquartile range (IQR), and nonparametric analysis methods were applied. Based on the sample analysis results, the data from the nest building test were analyzed using the Mann–Whitney U test, those from the open field test were analyzed with Student’s *t*-test, and the cytokine quantification data (due to the small sample size) were evaluated using the Fisher–Pitman exact permutation test (all possible permutations). Repeated measures ANOVA (RMANOVA) and Duncan’s post hoc testing were used for data analysis in tests with multiple trials (odor discrimination, learning and recall stages of the water maze, passive avoidance tests). When the *p*-value was less than 0.05, differences were considered statistically significant. In cases where *p* < 10^−16^, *p*→0 was indicated.

## 3. Results

### 3.1. Irradiation Improved Welfare of 5xFAD Mice

**Figure 2:**
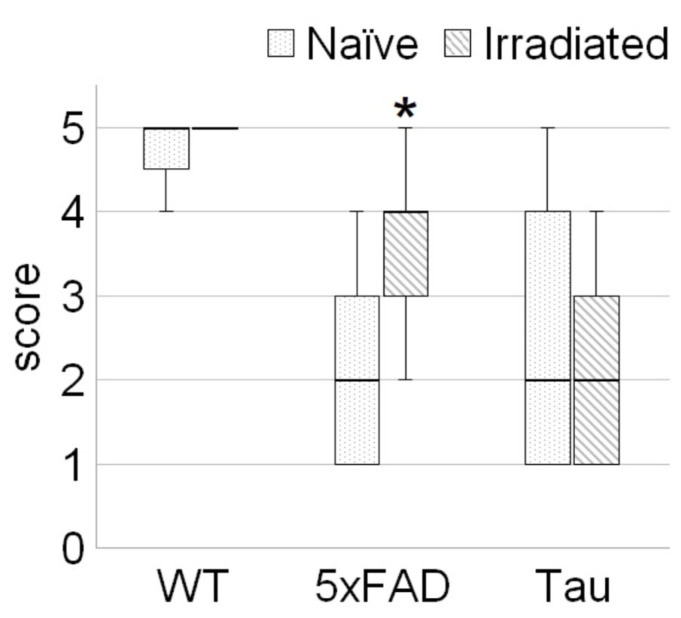
Nest building test. Data are presented as median (horizontal boldface line) ± IQR (upper and lower sides of the “box”), with the corresponding bars indicating the minimum and maximum values of the trait in the sample; *n*_(WT)_ = 7, *n*_(WT+R)_ = 6, *n*_(5xFAD)_ = 9, *n*_(5xFAD+R)_ = 9, *n*_(Tau)_ = 11, *n*_(Tau+R)_ = 8. Asterisk (*) indicates statistically significant differences between the control and the irradiated group (* *p* < 0.05; Mann–Whitney U test).

There was no significant effect of irradiation on the nest building of WT and Tau mice. Statistically significant differences were found only when evaluating the nests of the 5xFAD groups (Figure 2). The irradiated 5xFAD + R mice (nonnormal distribution: W = 0.84, *p* = 0.045; Shapiro–Wilk test) were characterized by higher-quality nest construction—the median score was 2-fold (U = 13.5, *p* = 0.02) higher compared to that of the intact 5xFAD mice (nonnormal distribution: W = 0.78, *p* = 0.012; Shapiro–Wilk test).

### 3.2. Irradiation Caused Anxiolytic Effects in Tau Mice and Stimulated Orientation and Exploratory Behavior in Tau and WT Mice

**Figure 3:**
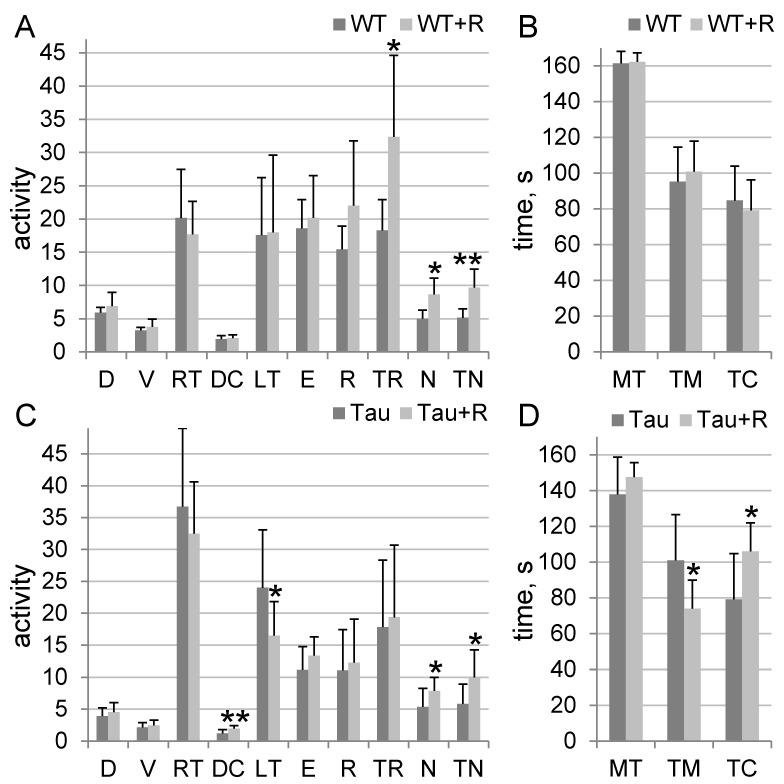
Open field test. Bar charts show mean + SD; *n*_(WT)_ = 7, *n*_(WT+R)_ = 6, *n*_(Tau)_ = 11, *n*_(Tau+R)_ = 8. D—distance traveled, m; V—speed, cm/s; RT—rest time, s; DM—distance traveled in margin, m; DC—distance traveled in arena center, m; LT—latency time, s; E—center arena entries; R—number of rearing; TR—total rearing duration, s; N—number of hole-poking; TN—total hole-poking duration, s; MT—time in motion, s; TM—time spent at the margins, s; TC—time spent at arena center, s. Asterisks (*) indicate statistically significant differences between the groups (* *p* < 0.05, ** *p* < 0.01; Student’s *t*-test).

Combined irradiation had an effect on WT mice activity: WT + R mice showed a higher rearing duration, number of hole-poking, and total hole-poking duration by 77% (*p* = 0.04), 73% (*p* = 0.01), and 88% (*p* = 0.009), respectively, compared to WT mice (Figure 3A,B). Irradiation had a more significant effect on Tau mice activity: Tau + R mice exhibited a higher distance traveled in the arena center, spent more time in the center, showed a higher number of hole-poking, and spent a longer time hole-poking, showing increases of 63% (*p* = 0.008), 34% (*p* = 0.01), 47% (*p* = 0.04), and 72% (*p* = 0.036), respectively, compared to Tau mice. They also had a lower first exit time to the arena center (latency time) and spent less time at the margins, showing decreases of 31% (*p* = 0.04) and 27% (*p* = 0.01), respectively, compared to the Tau group of mice (Figure 3C,D). In contrast, no significant effect of irradiation on 5xFAD mice activity in the open field was found.

### 3.3. Irradiation Improved Ability to Discriminate Odors in 5xFAD Mice

**Figure 4:**
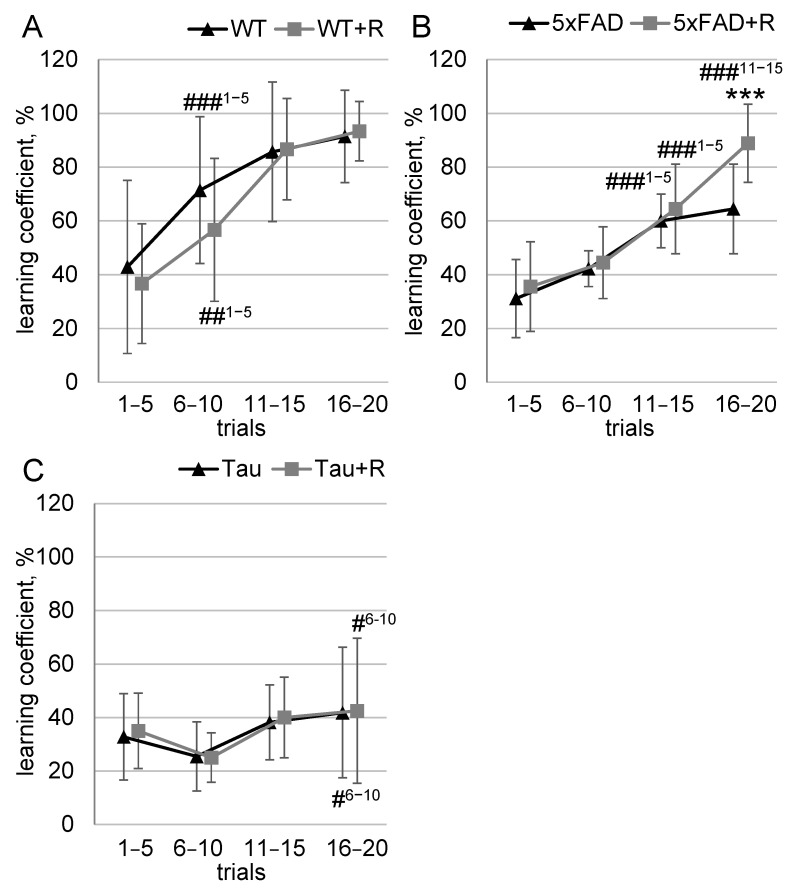
Odor discrimination test. Data presented as mean ± SD; *n*_(WT)_ = 7, *n*_(WT + R)_ = 6, *n*_(5xFAD)_ = 9, *n*_(5xFAD+R)_ = 9, *n*_(Tau)_ = 11, *n*_(Tau+R)_ = 8. Asterisks (*) indicate statistically significant differences between the groups (*** *p* < 0.001; Duncan’s post hoc test). Hashes (#) indicate statistically significant differences within a group compared to the point of analysis indicated by the superscript index (# *p* < 0.05, ## *p* < 0.01, ### *p* < 0.001; Duncan’s post hoc test).

The WT and WT + R groups showed excellent learning dynamics (F_3,33_ = 52; *p* = 10^−12^), whereas no effect of irradiation was detected. During the period of trials 6–10, both WT and WT + R mice showed an increase in learning coefficient by 1.7-fold (*p* = 0.0006) and 1.5-fold (*p* = 0.007), respectively, compared to the period of trials 1–5 (Figure 4A).

For 5xFAD mice, both the learning dynamic (F_3,48_ = 47; *p* = 10^−14^) and the irradiation effect (F_3,48_ = 3.4; *p* = 0.025) reached statistical significance. A significant increase in the learning coefficient occurred during the period of trials 11–15, when 5xFAD and 5xFAD + R mice showed increases of 1.9-fold (*p* = 5 × 10^−5^) and 1.8-fold (*p* = 5 × 10^−5^), respectively, compared to the period of trials 1–5. At the same time, during trials 16–20, 5xFAD + R mice showed a 1.38-fold (*p* = 0.0006) increase in the number of correct feeder choices compared to 5xFAD mice. Moreover, only the 5xFAD + R mice showed a significant 1.38-fold (*p* = 0.0002) increase in learning rate between trials 11–15 and 16–20 (Figure 4B).

Although the Tau and Tau + R groups of mice showed a significant change in learning dynamics (F_3,51_ = 3.8; *p* = 0.016), the learning coefficient of these groups did not exceed the target threshold of 50%, and no irradiation effect was detected. During the period of trials 16–20, the Tau and Tau + R mice showed an increase in learning coefficient of 1.6-fold (*p* = 0.02) and 1.7-fold (*p* = 0.04), respectively, compared to the period of trials 6–10 (Figure 4C).

### 3.4. Irradiation Enhanced Spatial Learning and Long-Term Spatial Memory of WT and Tau Mice

**Figure 5:**
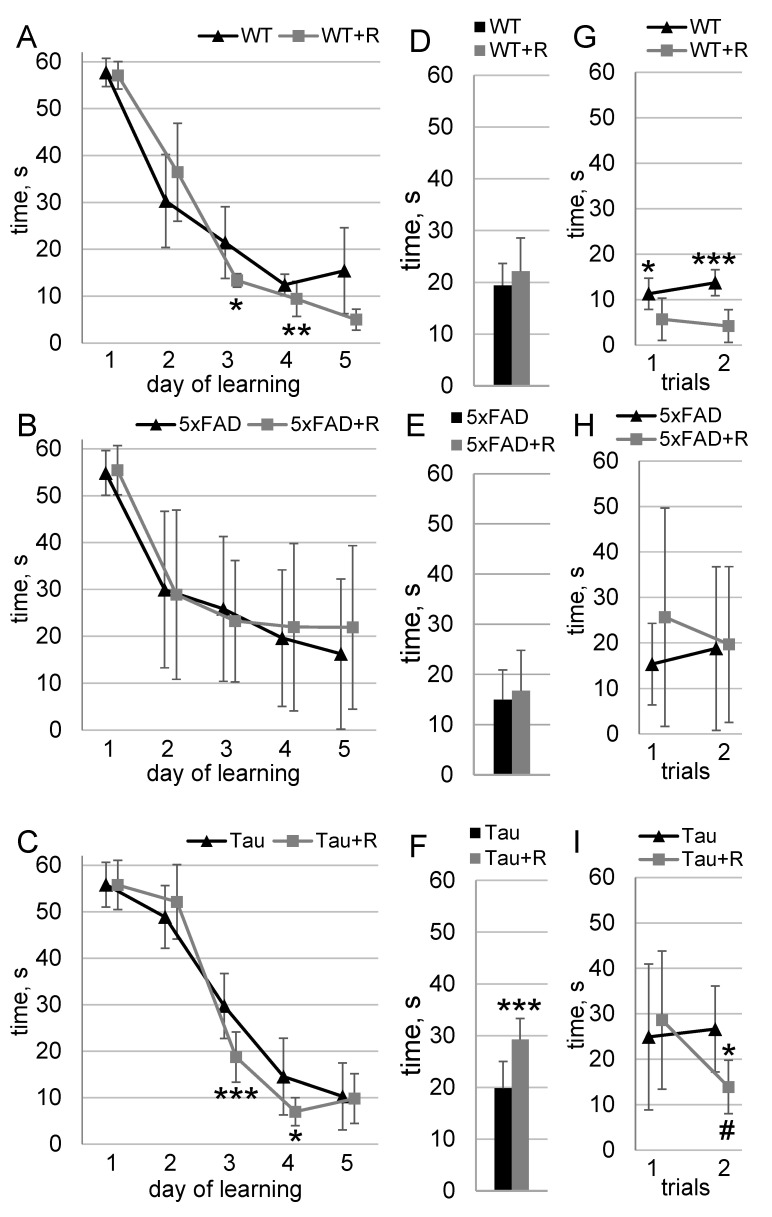
Morris water maze. Data presented as mean ± SD; *n*_(WT)_ = 7, *n*_(WT+R)_ = 6, *n*_(5xFAD)_ = 9, *n*_(5xFAD+R)_ = 9, *n*_(Tau)_ = 11, *n*_(Tau+R)_ = 8. (**A**–**C**) Learning dynamics; asterisks indicate statistically significant differences between the groups (* *p* < 0.05, ** *p* < 0.01, *** *p* < 0.001; Duncan’s post hoc test). (**D**–**F**) Probe test; asterisks indicate statistically significant differences between the groups (*** *p* < 0.001; Student’s *t*-test). (**G**–**I**) Recall spatial memory 1 week later final learning; asterisks (*) indicate statistically significant differences between the groups (* *p* < 0.05, *** *p* < 0.001; Duncan’s post hoc test), and hashes (#) indicate statistically significant differences within a group compared to the first trial (# *p* < 0.05; Duncan’s post hoc test).

The results of learning in the Morris water maze are shown in Figure 5. The WT and WT + R groups showed good learning dynamics (F_4,44_ = 123; *p*→0); however the influence of the radiation factor on the learning dynamics was also noted (F_4,44_ = 3,3; *p* = 0,02). On days 3 and 5 of testing, WT + R mice exhibited platform search times that were, respectively, 53% (*p* = 0.03) and 63% (*p* = 0.009) shorter compared to those of WT mice (Figure 5A). The test without the platform showed no statistically significant difference (Figure 5D). After 7 days, there was a significant irradiation effect in the recall phase (F_1,11_ = 29; *p* = 0.0002). WT + R mice took 50% (*p* = 0.01) and 70% (*p* = 0.0002) less time to find the platform on the first and second trials of the test, respectively, compared with WT mice (Figure 5G).

For the 5xFAD and 5xFAD + R groups, sufficient learning dynamics were observed (F_4,68_ = 26; *p* = 10^−12^). No significant differences were found between the groups during the learning process, the test without the platform, or the recall phase (Figure 5B,E,H).

For the Tau and Tau + R groups, good learning dynamics were observed (F_4,68_ = 270; *p*→0); however, the influence of the radiation factor on the learning dynamics was also noted (F_4,68_ = 4.9; *p* = 0.001). On days 3 and 4 of testing, Tau + R mice showed 40% (*p* = 0.0005) and 52% (*p* = 0.02) shorter platform search times, respectively, compared to Tau mice (Figure 5C). In the test without the platform, Tau + R mice spent 48% (t = 4.3; *p* = 0.0005) more time in the quadrant with the platform (Figure 5F). After 7 days, there was a significant effect of exposure on the recall dynamics during the recall phase (F_1,17_ = 5.1; *p* = 0.036). Tau + R mice spent 48% (*p* = 0.045) less time searching for the platform on the second trial of the test compared to Tau mice. During the second test trial, Tau + R mice demonstrated a 52% (*p* = 0.016) reduction in platform search time compared to the first trial. In contrast, no significant reduction in platform search time was observed for Tau mice between the first and second test trials (Figure 5I).

### 3.5. Irradiation Had No Effect on Fear Memory

All groups of tested mice showed a significant increase in time on the platform at the recall stage of the step-down-type passive avoidance test. No effect of IR exposure was detected.

### 3.6. Irradiation Drastically Influenced Cytokine Content in WT and Tau Mice, and to a Much Lesser Extent in 5xFAD Mice

**Figure 6:**
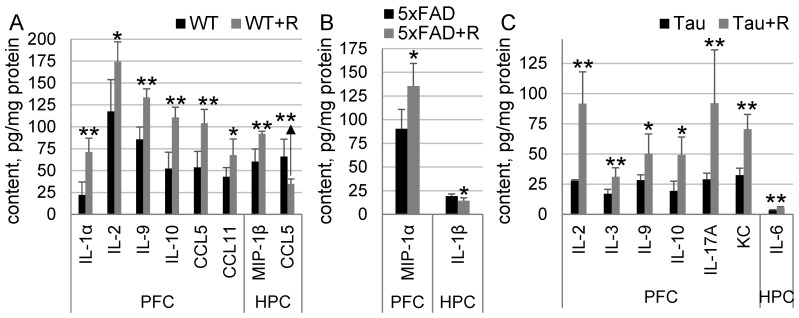
Quantification of cytokines. Data presented as mean ± SD; *n*_(WT)_ = 5, *n*_(WT+R)_ = 5, *n*_(5xFAD)_ = 5, *n*_(5xFAD+R)_ = 5, *n*_(Tau)_ = 5, *n*_(Tau+R)_ = 5. PFC—prefrontal cortex; HPC—hippocampus. Asterisks (*) indicate statistically significant differences between the groups (* *p* < 0.05, ** *p* < 0.01; Fisher–Pitman permutation test).

Increased content of the following cytokines was detected in the PFC of WT + R mice compared to WT mice: IL-1α, by 220% (*p* = 0.008); IL-2, by 48.5% (*p* = 0.03); IL-9, by 56% (*p* = 0.008); IL-10, by 112% (*p* = 0.008); CCL5, by 94% (*p* = 0.008); and CCL11, by 112% (*p* = 0.03). A 52.7% (*p* = 0.008) increase in MIP-1β and a 48% (*p* = 0.008) decrease in CCL5 were found in the HPC of WT + R mice compared to WT mice (Figure 6A).

Irradiation had limited effects on cytokine content in 5xFAD mice. There was a 50% (*p* = 0.015) increase in MIP-1α content in the PFC and a 25% (*p* = 0.02) decrease in IL-1β content in the HPC of 5xFAD + R mice compared to 5xFAD mice (Figure 6B).

Increased content of the following cytokines was found in the PFC of Tau + R compared to Tau mice: IL-2, by 229% (*p* = 0.008); IL-3, by 82% (*p* = 0.008); IL-9, by 77% (*p* = 0.02); IL-10, by 155% (*p* = 0.02); IL-17A, by 218% (*p* = 0.008); and KC, by 117% (*p* = 0.008). A 62% (*p* = 0.008) increase in IL-6 content was found in the HPC of Tau + R mice compared to Tau mice (Figure 6C).

## 4. Discussion

Based on literature data [37,38], we performed IR exposure of Tau P301S mice at the early stage of pathology, behavioral testing at the early symptomatic stage of tauopathy, and a postmortem study of cytokine content at the terminal stage of tauopathy in an effort to avoid natural decline in the livestock groups of mice. Irradiation of 5xFAD mice was performed at the onset of the first episodes of AD-related behavioral symptoms [39,40]. The animals underwent a battery of behavioral tests during the early symptomatic stage of the pathology [41,42], and euthanasia was performed at the same stage. In choosing the time points for analysis, we relied on data indicating that the beneficial effects of IR in neurodegenerative disease conditions in rodents are evident at 1.5–2.5 months [41,42] and may last up to 7 months after irradiation [29], as well as on earlier data obtained using an identical exposure model [25]. Different time points of analysis for 5xFAD and Tau P301S mice are associated with the specificity of neurodegeneration progression. C57Bl/6 mice were used to evaluate the effects of combined IR exposure at the same doses and composition on naïve mice, as it had previously been studied exclusively in Wistar rats [29,43].

Ethological analysis showed that combined IR exposure enhanced spatial learning and improved long-term spatial memory of Tau P301S mice. Moreover, irradiation enhanced orientation and exploratory behavior and caused an anxiolytic effect. IR had a similar effect on C57Bl/6 mice, except for the anxiolytic action. The obtained data are in good agreement with studies showing IR-induced (at a relevant dose) enhancement of both spatial learning and exploratory behavior in healthy rats [29,43,44] and mice [45], except for the anxiolytic effect of IR, which we discovered for the first time in neurodegenerative disease conditions. On the contrary, while having no effect on spatial memory and learning in 5xFAD mice, irradiation resulted in improved performance in the nest building test, indicating an improvement in general welfare and the functional integrity of the sensory and motor systems [46]. Moreover, although both intact and irradiated 5xFAD mice showed good learning dynamics (>50% correct trials at the end of learning), irradiation led to significant improvement in the odor discrimination test. Since the acquisition of olfactory discrimination depends mainly on the functioning of the olfactory bulb, olfactory cortex, and HPC [47,48,49], the test results may indicate improved functional integration of these structures in irradiated 5xFAD mice. Thus, we did not discover any additive or synergistic effects of IR exposure and AD-related pathology. On the contrary, we discovered several IR-induced phenomena that could be considered beneficial in neurodegenerative conditions.

It is known that acute IR exposure is accompanied by a neuroinflammatory response, including upregulation of brain cytokines, which can persist for up to 9 months [50,51,52,53]. Despite some disagreements [54], neuroinflammation is generally defined as a CNS condition accompanied by activation of microglia and astrocytes. Moreover, activated microglia are characterized by increased expression of both pro- and anti-inflammatory cytokines [55], which is often regarded as a marker of microglial activation and neuroinflammation [14,56,57]. We found that irradiated Tau P301S and C57Bl/6 mice are characterized, to varying degrees, by elevated levels of both pro- and anti-inflammatory cytokines. Thus, we hypothesize that IR leads to microglial activation in Tau P301S and C57Bl/6 mice and these changes are associated with behavior benefits. There are several possible explanations for the enhanced cognitive abilities observed alongside increased cytokine levels. Firstly, IR-induced behavioral effects can be realized through the direct enhancement of neuroinflammation by activating microglia, which entails the clearance of both pathological protein aggregates caused by neurodegenerative conditions and cellular debris resulting from IR-induced apoptosis [50,51]. Indeed, despite the upregulation of pro- and even anti-inflammatory cytokines being implicated in the progression of neurodegeneration, cytokines such as IL-2 [58], IL-3 [59], IL-6 [60], IL-9 [61], IL-10 [62], IL-17A [63], and KC [64] have also been shown to enhance microglial phagocytosis and ameliorate AD-like pathology. It has been shown that IL-2 level is positively correlated with a reduction in pathological protein aggregates in a new transgenic model of neurodegenerative disease (crossbreeding 5xFAD and Thy-Tau22 mice) [65]. IL-2 treatment was observed to be associated with astrocytic recruitment around Aβ plaques and rescue hippocampal spatial memory impairments, synaptic defects, and Aβ pathology in APP/PS1 transgenic mice [66]. Moreover, IL-2 is important for spatial learning and memory [67]. IL-3 elicits programming of microglia, endowing them with an acute immune response program, enhanced motility, and the capacity to cluster and clear aggregates of Aβ and tau. These changes restrict AD pathology and enhance orientational and exploratory behavior, as well as spatial learning, in 5xFAD mice [68]. Importantly, these data are in good agreement with the enhanced exploratory behavior we found in irradiated Tau P301S mice. Genetically induced overexpression of IL-6 in APP transgenic TgCRND8 mice results in marked suppression of Aβ deposition, which the authors suggest occurs through enhanced microglia-mediated phagocytosis of Aβ aggregates [69]. Along with this, brain IL-17A overexpression decreased soluble Aβ levels in the HPC and cerebrospinal fluid, drastically reduced cerebral amyloid angiopathy, and caused anxiolytic and spatial memory-enhancing effects in an AD mouse model [70]. Additionally, it has been reported that spatial learning improvement induced by voluntary exercise was accompanied by an increase in KC chemokine levels in the HPC of aged Tg2576 mice [71]. Secondly, IR-induced behavioral effects can be realized through the neuroprotective properties of cytokines and their involvement in the modulation of cognitive processes. It is important to note the essential role of microglia in synapse pruning and neuronal network maturation, processes modulated by cytokines [12]. It has been shown that IL-1α might facilitate memory extinction [72], and a slight increase in brain IL-1 levels can improve HPC-dependent memory [73]. IL-2 was shown to support the survival and enhanced neurite extension of cultured hippocampal neurons, indicating its neurotrophic function [74]. IL-3 has similar neurotrophic functions: both humans and mice studies have demonstrated the crucial role of IL-3 in expanding and maintaining the neural progenitor pool and the number of surviving neurons [75]. At the same time, IL-3 has a protective role against Aβ-induced cell death [76]. Moreover, IL-3 significantly reduced Aβ-promoted neurite degeneration in primary cortical neuronal cells by modulating microtubular dynamics and preventing tau cleavage and hyperphosphorylation [77]. Increased levels of another cytokine, IL-6, have been shown to promote neurite outgrowth [78]. Blockade of CCL5 signaling also results in enhanced fear memory consolidation [79]. In contrast, the research using knock-out mice showed that CCL5 actions on glucose aerobic metabolism are critical for mitochondrial function, which contributes to hippocampal spine and synapse formation, thereby improving learning and memory [80]. Another study showed that CCL5 has a neuroprotective effect under oxidative stress through the activation of glutathione peroxidase-1 expression [81]. Interestingly, the effect of IR on CCL5 content was multidirectional in the PFC and HPC of C57Bl/6 mice. Another chemokine, CCL11, may be involved in the realization of IR-induced proliferation, as described previously [82,83]. CCL11 promotes the migration and proliferation of neural progenitor cells and plays a crucial role in neuroregeneration after neonatal hypoxic–ischemic brain injury [84]. As far as we know, the role of MIP-1β in physiological conditions remains largely unstudied. We believe that an increase in MIP-1β levels may be necessary to clear the nervous system of cellular debris resulting from IR-induced apoptotic cell death [85].

Of particular interest is the increase of IL-9 and IL-10 with anti-inflammatory properties in both irradiated Tau P301S and C57Bl/6 mice. In a postmortem study of the brains of patients with multiple sclerosis, it was shown that IL-9 reduces the activation state and promotes the anti-inflammatory properties of macrophages. The authors suggest that this mechanism may contribute to the beneficial effects of IL-9 that are observed in multiple sclerosis [86]. IL-9 also possesses neuroprotective properties, inhibiting apoptosis in the newborn neocortex [87]. IL-10 is considered to be an important anti-inflammatory modulator of glial activation, preventing inflammation-mediated neuronal degeneration under pathological conditions. IL-10 can directly protect cortical neurons in culture following exposure to oxygen-glucose deprivation or glutamate toxicity [88]. Moreover, a study using a knock-out mouse line demonstrated that the loss of IL-10 activates microglia, enhances IL-6, and leads to hyperphosphorylation of tau on AD-relevant epitopes in response to acute systemic inflammation [89].

Surprisingly, IR had a limited effect on the content of analyzed cytokines in 5xFAD mice. We hypothesized that this may be due to one of the physiological roles of Aβ: the antioxidant function, which was predicted earlier [90,91]. Indeed, one of the primary effects of IR is the generation of free radicals and their induced oxidative stress [30,92], which leads to neuroinflammation [53,93]. In contrast, antioxidants exert anti-inflammation and general radioprotective effects [94]. Thus, the possible free radical scavenging activity of Aβ, which presents as Aβ plaques in the brain of 5xFAD mice in much higher amounts than in C57Bl/6 [95] and probably in Tau P301S mice, may limit those effects of IR that are associated with free radical generation, including reducing the severity and duration of neuroinflammation [53]. At the same time, the decreased IL-1β content in the HPC and increased MIP-1α content in the PFC observed by biochemical analysis are not inconsistent with the behavioral phenotype of irradiated 5xFAD mice. Thus, despite the critical importance of IL-1β for spatial learning in 3-month-old mice, this importance is not confirmed in 6-month-old mice [72]. Moreover, elevated IL-1β production is associated with AD pathology, and its reduction may alter brain inflammatory responses and alleviate cognitive deficits [96]. Although elevated MIP-1α is also associated with neurodegenerative pathology, including AD [97], MIP-1α may enhance monocyte differentiation into macrophages and their migration through the blood–brain barrier, which could favor Aβ phagocytosis in the early stages of AD [98].

It is important to take into account that our findings on the link between behavioral benefits and increased cytokine levels only apply if we assume that the effects of cytokines depend on the state of the central nervous system (CNS) and can be both neurodamaging and neuroprotective. Indeed, this assumption is supported by the literature, which indicates that cytokines can have both neurodamaging and neuroprotection properties depending on many factors [99,100]. Furthermore, considering the multitargeting nature of IR exposure on the CNS [30], improvements in AD-like symptoms or pro-cognitive effects of irradiation in a healthy brain may be attributed to a complex interplay of factors.

Our study has potential limitations. Although a statistically significant enhancement of spatial learning was found in the irradiated C57Bl/6 and Tau P301S mouse lines, the enhancement was not particularly large and was only detected on days 3 and 4 of the learning. Moreover, the probe test confirmed this enhancement only in the Tau P301S mice. More convincing results were obtained when long-term spatial memory was assessed. In this pilot study, we clearly detected increased cytokines and behavioral changes in irradiated animals but cannot prove a link between them. Our proposed scenarios of possible links are speculative and can only be confirmed or refuted by future studies.

## 5. Conclusions

The obtained results suggest that combined IR (γ-ray whole-body pre-irradiation and acute ^12^C head irradiation) leads to a selective increase (in C57Bl/6 and Tau P301S mice) or limited “re-balancing” (in 5xFAD mice) of interleukin/chemokine content, primarily in the PFC and, to a much lesser extent, in the HPC. This modulating effect of IR on microglial activity is associated with cognitive and non-cognitive behavioral benefits in both naïve C57Bl/6 mice and in transgenic models of AD (Tau P301S and 5xFAD mice). Thus, combined IR has proven to be an effective tool for controlling microglial activity, inducing a persistent effect that may be potentially useful, given the other effects of IR, in the treatment of neurodegenerative diseases, particularly AD.

## Data Availability

“Combined ionizing radiation caused cognition and non-cognition behavior benefits and modulated innate immune system activity in wild-type and Alzheimer’s-like transgenic mice”, Mendeley Data, V1, https://doi.org/10.17632/5crf4kj8xw.1.
